# Maternal health services utilization and maternal mortality in China: a longitudinal study from 2009 to 2016

**DOI:** 10.1186/s12884-020-02900-4

**Published:** 2020-04-15

**Authors:** Pengyu Zhao, Xueyan Han, Lili You, Yu Zhao, Li Yang, Yuanli Liu

**Affiliations:** 1 School of Public Health, Chinese Academy of Medical Science & Peking Union Medical College, No.5 Dongdansantiao, 100730 Beijing, People’s Republic of China; 2 Nursing Department, Peking University First Hospital, No.8 Xishiku Street, Xicheng District, 100034 Beijing, People’s Republic of China

**Keywords:** Maternal health management, Service utilization, Maternal mortality, Basic public health service, China

## Abstract

**Background:**

The Chinese government introduced the maternal health services as part of the Basic Public Health Service (BPHS) project in 2009. While China has made progress in reducing maternal mortality, the longitudinal association between the utilization rate of the maternal health services of the BPHS project and such reduction was rarely evaluated with robust methods.

**Methods:**

We conducted a longitudinal study on the maternal health service utilization rates of the BPHS project and the maternal mortality ratios (MMR) in mainland China and its 31 provincial regions. The data were extracted from the National Health Statistic Yearbooks (2009–2016). Panel data models were used to evaluate the association between the utilization rate and the MMR after adjusting for available covariates on healthcare resources. Stepwise regression models were used to gauge the direction and magnitude of omitted variable bias.

**Results:**

It was found that the service utilization rate increased from 79.24% in 2009 to 91.67% in 2016, while MMR decreased from 30.90 to 17.88 per 100,000 livebirths at the national level. The results of the fixed effects panel data model revealed that the utilization rate was associated with MMR deduction continuously. With every 1% increase in utilization rate, the maternal death would decrease by 0.35 per 100,000 livebirths after having the health resources variables adjusted.

**Conclusions:**

The utilization of maternal health services increased continuously after the introduction of BPHS project in 2009 and MMR went downward from then on. The utilization of maternal health services did have significant association with MMR and could reduce MMR effectively. Such improvement could be attributed to the fact that this program was designed to serve the targeted population throughout the continuum of maternal care and the government’s rigorous efforts in stressing equality and standard care in program implementation.

## Background

Approximately 800 women die each day due to preventable causes related to pregnancy and childbirth according to the World Health Organization (WHO) report [1], indicating that the maternal mortality is still a worldwide public health challenge. The international community attached great importance to the maternal health and made it an important part of both the Millennium Development Goals (MDGs) [2] in 2000 and the Sustainable Development Goals (SDGs) [3] in 2015. It had been noted that 99% of the maternal deaths occurred in developing countries [1]. The main causes of maternal deaths were found to be obstetric hemorrhage, sepsis, unsafe abortion, hypertensive disorders, and obstructed labor, which were mostly preventable [4], and could be averted if all women had access to timely health services and interventions for the prevention and treatment of pregnancy and birth complications [5]. It was also reported that the high maternal mortality ratio (MMR) was associated with inadequate and poor-quality maternal health services from the global perspective [6]. Therefore, it has been widely recognized that maternal health service programs, which generally included antenatal healthcare, health education, mental health management, skilled delivery attendant, and postnatal healthcare [7–10] should be valued, carefully designed and vigorously evaluated [11, 12]. WHO have announced that the antenatal healthcare should include at least 4 visits [13] to health facilities for an uncomplicated pregnancy and at least 3 postnatal care visits [14] are recommended for all mothers and newborns.

China had designed and implemented policies to promote maternal and child health since the 1990s. In 2000, the “Reducing Maternal Mortality and Eliminating Neonatal Tetanus” program was introduced and the New Cooperative Medical Scheme (NCMS) began to extend the healthcare insurance to the rural population in 2003 [15]. In order to further accelerate the fulfillment of MDGs and improve equality in health services, the Chinese government launched the BPHS project in 2009 [16]. The whole BPHS project contained 9 service categories [17], and maternal care program was one category in the whole project. The maternal health services required by the BPHS project mainly included the establishment of maternal health records, antenatal visits and postnatal visits performed by primary care medical staff at the community health centers or township health centers [18]. It was made clear that the maternal health program of the BPHS project aimed at providing services throughout the continuum of maternal care. The utilization rate of the maternal health program was introduced to measure how many eligible pregnant women were successfully received all the services specified by the program protocol. Although several other maternal health service programs had been accessed by previous studies, they tended to focus on services provided at a certain stage of maternal care continuum, such as antenatal [19] or postpartum stage. There were few studies discussed the association between maternal death and the maternal service utilization spanning the antenatal to post-natal periods. Furthermore, the BPHS project was designed and implemented with uniform standard in all 31 provinces in mainland China since 2009, with the determination that every pregnant woman should be able to receive these services in the same quantity and quality regardless of their residency, social-economic status and age. The maternal health intervention of this scale warranted detailed investigation. In addition, the statistical methods used in the previous studies were mostly univariate correlation analyses [20] or linear regressions [21], which may not be effective enough to adjust the time-invariant in the longitudinal data. Panel data models, on the other hand, could be more suitable to analyze the cross-sectional samples with multiple time points [22, 23].

The maternal health services, introduced as a part of the BPHS project of China, had been implemented for a decade and it was high time to assess whether these maternal services were connected to the decrease in maternal deaths. Therefore, this study aimed to explore the trends of change in the maternal health services utilization rate and the maternal mortality over time, and to analyze the longitudinal association between the service utilization and the maternal mortality.

## Methods

### Study design

The study was based on the panel data of maternal health services utilization rate and maternal mortality at the provincial level from 2009 to 2016. The dependent variable in this study was the MMR. The explanatory variable of interest was the maternal health service utilization rate, and the healthcare resources variables were used for risk-adjustment. All the data in this study were extracted from the relevant sections in the National Health Statistic Yearbook of the corresponding years.

### Data collection

The MMR, the maternal health service utilization rates and the health resource data of the mainland China and its 31 provincial regions were collected by the Chinese government and reported in the National Health Statistic Yearbook.

#### The maternal health service utilization rate

Table 1 listed the maternal health services required by the *Maternal Health Care Specification issued by People’s Republic of China* of the BPHS project in detail [24]. The pregnant women who received all these services were counted in the numerator of the maternal health utilization rate, and all the pregnant women whose pregnancy ended in live birth(s) were counted in the denominator of the utilization rate.

**Table 1:** Maternal health services protocol specified in the BPHS project

Service items	Ante-natal Services			Postnatal services	
	1st trimester	2nd trimester	3rd trimester	7 days postpartum	42 days postpartum
Number of visits					
Minimum number of visits	1	2	2	1	1
Required services					
Inspection on family history	√				
Inspection on previous diseases	√				
Gynecologic examinations	√	√	√		√
Routine blood test	√	√	√		√
Blood pressure	√	√	√		√
Routine urine test	√	√	√		√
Liver function tests	√	√	√		√
Kidney function tests	√	√	√		√
Hepatitis B examinations	√				√
Syphilis test and treatment	√				√
HIV test and counselling	√				√
Establishment of maternal health record	√				
Guidance of lifestyle, nutrition and mental health	√	√	√	√	√
Health education on spontaneous labor			√		
Health education on breast feeding			√	√	
Neonatal care instructions				√	√

Notes: This table summarized the requirements in the *Maternal Health Care Specification issued by People’s Republic of China* [24]

By definition, the maternal health service utilization rate required that the pregnant women who had accept all the antenatal and postnatal maternal health services listed in Table 1, plus the perinatal services (sterilization during pregnancy, skilled birth attendant) to be counted in the numerator. The perinatal services were not included in the BPHS project but it was a required component in the utilization rate of the maternal health program in the BPHS project. The perinatal services had been kept at a high level in China since 2000, and changed very little from 2009 to 2016 (increased from 99.3 to 99.9%).

The primary care medical staff at the community health centers or township health centers would provide these services uniformly in same quantity and quality to guarantee the safety of pregnant women in all periods. They were also required to register and report on the completion of each maternal health services in order to calculate the utilization rate among other program indicators. These data were then submitted to the provincial government and finally to the Ministry of Health. The Ministry of Health would also organize experts to conduct sampling survey and supervision of maternal health services in every province so as to evaluate the project implementation. The Ministry of Health summarized and examined the data quality reported by the provinces based on their evaluation and then included them in the National Health Statistic Yearbook, which was published annually as public information [25].

#### The maternal mortality ratio

The official definition of maternal death of WHO was the death of a woman who was pregnant or within 42 days of pregnancy termination, irrespective of the duration and the site of the pregnancy, from any cause related to or aggravated by pregnancy or its management. Accidental or incidental causes related deaths of eligible women were not considered as maternal deaths [26]. This is the definition of maternal death in China as well, and the unit of dependent variable was the maternal death per 100,000 livebirths. To monitor the maternal death in China, the Chinese government set up the National Maternal Mortality Surveillance System (NMMSS) in 1989. The sampling unit of the system was at the county (district) level. A total of 336 surveillance spots (126 urban areas and 210 rural areas), covering 31 provinces, were selected to record the changes in MMR and the main cause of the maternal death. Livebirths and maternal death data were collected by trained officials and verified by government administrators [11] and then included in the National Health Statistic Yearbook.

#### Health resource data

The structural data on health resource allocation (service fee per capita, doctors per thousand population, urban-rural doctor ratio, nurses per thousand population, urban-rural nurse ratio, inpatient beds per thousand population, urban-rural inpatient beds ratio) from 2009 to 2016 of each province were extracted from the National Health Statistic Yearbook. The service fee per capita was the total financial input of the whole nation or a certain province divided by the population covered by the funding. The data on health resources allocation were collected to serve as covariates in the model. Variables such as insurance coverage, number of medical staff (e.g. gynecologists, obstetricians, or specialized nurses) were not included in this study due to the lack of official statistics.

### Statistics analyses

We applied descriptive analyses to demonstrate the changes of the service utilization rate, the MMR and the health resource allocation variables on the national and provincial level from 2009 to 2016. As for the panel data models, pooled effects model, fixed effects model and random effects model were all applied in this study. Comparing to linear models, these panel data models were more effective in controlling confounding factors [27]. The F test and the Hausman-type test would be used for model selection [28]. The plm package of R software was used to conduct the panel model analyses. The years were treated as observation time points and each province was viewed as a cross section, and an 8*31 data matrix was constructed. The detailed data analysis process was as follows: the data was firstly fitted with the fixed effects model, random effects model and pooled effects model respectively. Then the F test was taken to choose between fixed effects and pooled effects specifications, and comparing the estimators under the null hypothesis of no significant difference between the two models. If this null hypothesis was rejected, then the more efficient fixed effects estimator was chosen. The selection for fixed and random effects specifications was based on Hausman-type test, if the null hypothesis of this test was rejected, a fixed effects model would be chosen [29]. Finally, according to the results, the reasonable panel data model would be chosen to elaborate the relationship between the service utilization rate and the MMR after controlling for the available health resources factors. The estimated coefficients would give the expected change in the dependent variable associated with a one-unit increase in a certain explanatory variable, while other variables were held constant. Stepwise regression models were applied to inspect the collinear effect and to gauge the direction and magnitude of omitted variable bias. We estimated four regression models for the dependent variable (the MMR). Model 1 was a univariate model including only utilization rate of maternal health services as the explanatory variable. Statistically significant coefficients on the utilization rate of maternal health services would provide evidence on the unadjusted effect of the utilization rate. Model 2 and 3 added explanatory variables capturing mediate factors suspected to channel the effects of utilization rate of maternal health services on the MMR. Model 4 included all the explanatory variables at the same time. Statistical analyses were conducted with R software (version 3.5.1) with the significance level of *p* < 0.05.

## Results

**Fig. 1:**
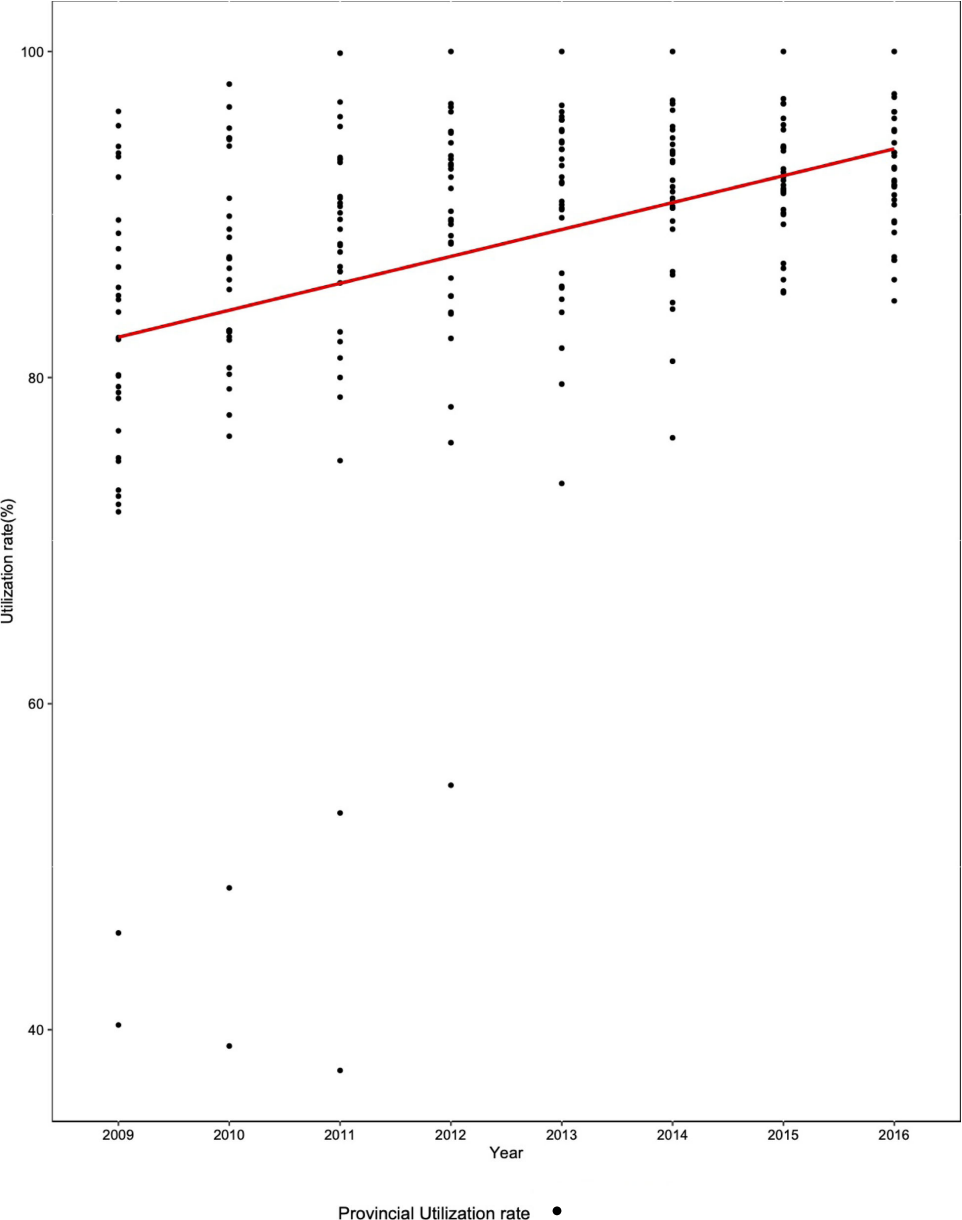
The variations and trend changes of provincial service utilization rate from 2009 to 2016

**Fig. 2:**
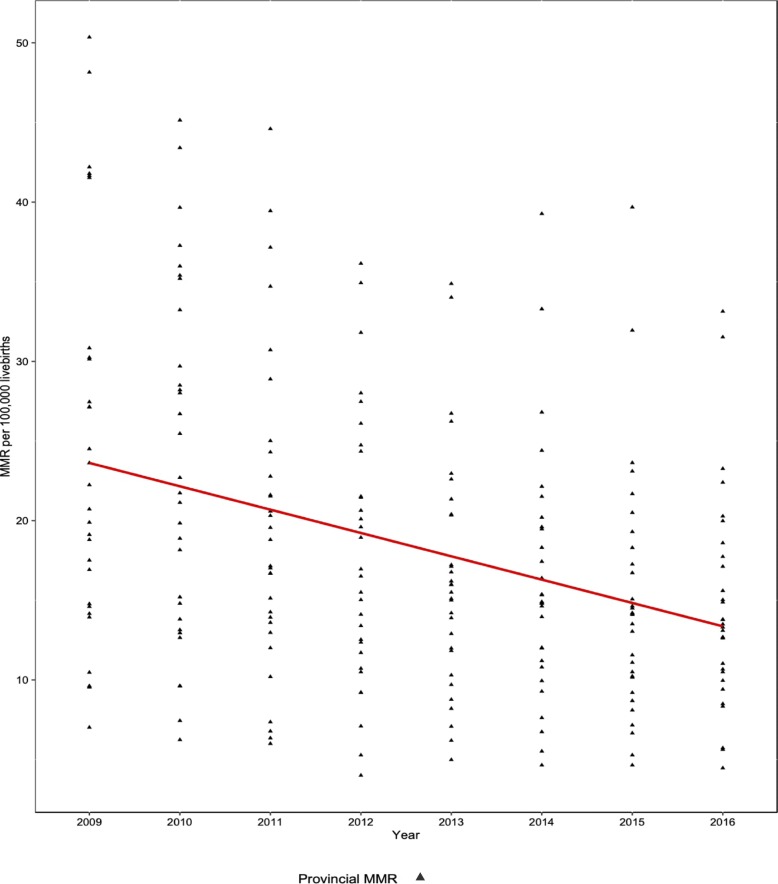
The variations and trend changes of provincial MMR from 2009 to 2016

It was found that the service utilization rate increased from 79.24% in 2009 to 91.67% in 2016, while MMR decreased from 30.90 to 17.88 per 100,000 livebirths at the national level. The variations and trend changes of provincial service utilization rates, the MMR as well as the information on healthcare resource allocation from 2009 to 2016 were shown in Figs. 1, 2 and Table 2. It was observed that there was a huge variation among provinces for all the variables. Over time, the gap of the service utilization rate had been narrowed from 63.00 in 2009 to 25.60 in 2016. Similarly, the provincial MMR had also been narrowed from 225.21 to 105.43 per 100,000 livebirths during the eight years of observation.

**Table 2:** The provincial variation of the variables from 2009 to 2016

Variable	2009	2010	2011	2012	2013	2014	2015	2016
MMR	7.02–232.23	6.24–174.78	6.00–180.74	4.01–176.12	4.99–154.51	4.65–108.86	4.65–100.92	4.47–109.9
Utilization rate	33.33–96.33	32.2–98.00	30.7–99.90	38.20–100.00	44.20–100.00	49.60–100.00	71.00–100.00	74.40–100.00
Service fee per capita	10.00–58.00	15.00–67.00	20.00–95.00	25.00–101.00	30.00–110.00	35.00–117.00	40.00–136.00	45.00–131.00
Doctors per thousand population	0.81–4.70	0.83–4.90	0.86–5.11	0.94–3.37	1.03–5.50	1.25–3.50	1.40–3.70	1.40–3.90
Urban-rural doctor ratio	1.51–4.55	1.33–6.53	1.33–6.55	1.37–5.42	1.48–5.90	1.49–5.73	1.59–5.86	1.06–5.91
Nurses per thousand population	0.69–4.95	0.68–5.34	0.71–5.68	0.56–3.84	0.76–6.36	0.85–4.11	1.00–4.40	1.20–4.50
Urban-rural nurse ratio	2.07–8.22	2.01–11.66	2.05–10.11	1.83–8.32	1.84–10.62	2.20–8.39	2.16–7.82	1.64–8.27
Inpatient beds per thousand population	2.22–6.80	2.33–6.80	2.77–7.55	2.72–5.89	3.53–6.06	3.75–6.22	4.02–6.37	4.21–8.03
Urban-rural inpatient beds ratio	1.55–3.77	1.06–4.14	1.18–4.14	1.10–9.59	1.10–4.40	1.46–3.87	1.52–4.09	1.52–4.11

Notes: The data represent the ranges of the variables across provinces

The results of relationships between utilization rate and MMR across 8 time points based on the panel data were fitted with fixed effects model, random effects model and pooled effects model respectively, and the F test and Hausman-type test were taken to the assessment of the fitting effects of the above models. The results of all three models were shown in Table 3. The result of the F test was F = 1.86 (*p* < 0.01), which indicated that the fixed effects model was more appropriate than the pooled effects model in this situation. The statistics of the Hausman-type test were 75.89 (*p* < 0.01), which meant the fixed effects model induced less estimated bias than random effects model. Hence, the fixed effects model was used to estimate the relationship between the utilization rate and MMR in this study.

**Table 3:** Results of relationship between utilization rate and MMR based on the panel data models

Variable	Fixed Effects Model			Random Effects Model			Pooled Effects Model		
	β	S.E	***p***-value	β	S.E	***p***-value	β	S.E	***p***-value
Intercept				65.74	9.24	< 0.001	61.40	9.52	< 0.001
Utilization rate	−0.35	0.09	< 0.001	− 0.49	0.09	< 0.001	− 0.75	0.10	< 0.001
Service fee per capita	−0.30	0.08	< 0.001	−0.13	0.08	0.10	0.19	0.09	0.05
Doctors per thousand population	−21.38	6.38	< 0.001	−9.49	5.79	0.10	13.92	5.11	< 0.01
Urban-rural doctor ratio	−11.69	6.45	0.052	−9.83	2.51	< 0.001	0.37	2.49	0.88
Nurses per thousand population	20.47	12.31	0.06	8.02	5.17	0.12	−16.39	5.06	< 0.01
Urban-rural nurse ratio	9.78	1.77	< 0.001	12.23	1.77	< 0.001	12.22	1.86	< 0.001
Inpatient beds per thousand population	−2.77	1.17	0.02	−1.07	1.17	0.36	2.40	1.29	0.06
Urban-rural inpatient beds ratio	−1.30	1.19	0.28	−2.33	1.29	0.07	−11.01	1.81	< 0.001
F statistics		40.92			24.24			36.12	
Overall significance of model		< 0.001			< 0.001			< 0.001	

The results of the fixed effect panel stepwise regression models were shown in Table 4. Model 1 indicated that utilization rate (β = − 0.69, *p* < 0.001) had negative effect on the MMR. Model 2 added doctors per thousand population, nurses per thousand population and inpatient beds per thousand population to the model. Both doctors per thousand population (β = − 3.99, *p* = 0.001) and inpatient beds per thousand population (β = − 4.21, *p* < 0.001) were associated with MMR. Model 3 added variables of urban-rural doctor ratio, urban-rural nurse ratio and urban-rural inpatient beds ratio. Urban-rural nurse ratio (β = 9.56, *p* < 0.001) was found to be associated with MMR. Model 4 included all the variables and revealed the utilization rate was associated with MMR and could reduce MMR continuously after adjusting for health resource and other variables. The results also suggested that variables such as service fee per capita, number of doctors per thousand population, inpatient beds per thousand population had significant negative relationship with MMR. However, the increase in urban-rural nurse ratio was found to be associated with the increased maternal mortality. The urban-rural doctor ratio, nurses per thousand population, urban-rural inpatient beds ratio was found to have no statistically significant association with the MMR.

**Table 4:** Fixed effect panel stepwise regression models of utilization rate and MMR

	Model 1		Model 2		Model 3		Model 4	
	β	*p*-value	β	*p*-value	β	*p*-value	β	*p*-value
Utilization rate	−0.69	< 0.001	−0.50	< 0.001	−0.45	< 0.001	−0.35	< 0.001
Doctors per thousand population			−3.99	0.001	−7.07	0.01	−21.38	< 0.001
Nurses per thousand population			1.58	0.73	8.50	0.053	20.47	0.06
Inpatient beds per thousand population			−4.21	< 0.001	−3.10	0.01	−2.77	0.02
Urban-rural doctor ratio					−10.71	0.05	−11.69	0.05
Urban-rural nurse ratio					9.56	< 0.001	9.78	< 0.001
Urban-rural inpatient beds ratio					−1.80	0.13	−1.30	0.28
Service fee per capita							−0.30	< 0.001
Adjusted R^2^	0.15		0.22		0.31		0.34	

## Discussion

This study found that after the introduction of BPHS project in 2009, the maternal health care utilization rate was increasing from 2009 to 2016 while the MMR decreased steadily on the national level. Furthermore, the gap of the utilization rate and MMR among provinces from 2009 to 2016 were narrowed sharply, which indicated that the accessibility and equality of maternal health services may also been improved. Panel data models revealed that the service utilization rate was negatively associated with the MMR after adjusting for available covariates on healthcare resource allocation. This paper pointed to the increase in maternal health service utilization after introducing the BPHS project in 2009 and the importance of the continuous strengthening of the accessibility and quality of maternal healthcare services since it was found to be connected to the decrease in maternal mortality in China.

This study found that the disparity among the provinces had decreased from 2009 to 2016, which may reflect the improvement in healthcare equity in China. Primary healthcare service equalization was one of the essential objectives of the BPHS project since its introduction in 2009. Since geographic variance in quantity and quality of maternal health services were observed between urban and rural areas before the implementation of BPHS project [30], the project required all maternal health services to be implemented in the same quantity and quality by specialized medical staff after professional training. From the central government perspective, they had increased the overall financial input annually [16], and had reformed the data collection process (from paper-based reporting to the application of healthcare information systems). However, since the provincial-level subsidies to the BPHS projects (additional to the national funding) varied among provinces, regional variation of per capita service fees still existed. In 2016, the per capita service fee varied from 45.00–131.00 RMB, indicating that China would need targeted and varied strategies to fund the BPHS project in each region.

It was observed in this study that the maternal health service utilization was associated with the deduction in the MMR after other healthcare resource covariates were adjusted. As described above, previous studies had demonstrated the negative correlation between antenatal care [7, 30] or postpartum care [31, 32] and maternal mortality, but rarely were there any studies focused on the relationship between maternal mortality and maternal health management from the antenatal to postpartum period. The results of this study stressed the importance of maternal health management across care continuity. Previous studies had noted that the shortage of health resources and well-trained health workforce had always been important indirect factors affecting maternal mortality [33, 34], which was consistent with the findings of this study. Moreover, the increasing in doctors per thousand population and inpatient beds per thousand population had a significant negative association with the MMR. Meanwhile, it had been widely accepted that the MMR in rural or urban areas was affected by socioeconomic status [35] in addition to the development of healthcare systems. In China, healthcare resources and human resources were concentrated in eastern coastal regions, and the number of doctors and nurses were much higher than it was in the remote and inland western regions [30], which might hamper the efforts of the maternal healthcare services in reducing MMR. Our results showed that urban-rural nurse ratio had a positive association with the MMR, which may indicate that the imbalanced the urban-rural nurse distribution may increase the likelihood of maternal deaths. It was also found that the service fee per capita had significant negative association with maternal mortality, which might suggest that the medical service could be improved with increased medical input. In addition, this study found no significant connection between the maternal mortality and indicators such as nurses per thousand population, urban-rural doctor ratio, and urban-rural inpatient beds ratio. The reason behind these findings might be the wide definition of these variables and the limitation of the sample size in this study. The panel data analyses that used county or individual level data would further enlarge the sample size and adding the statistical power to explore the association between maternal health services and maternal mortality.

One of the major strengths of this study was that instead of focusing on services provided during certain phases of maternal health care, we discussed the association between the utilization rate of maternal health service from antenatal to postpartum period and maternal mortality. Furthermore, the application of the panel data models avoided information loss of the data. Compared with the cross-sectional data, the panel data might reflect the optimal validity of data by arranging the cross-sectional data and time-series data together [22, 27].

This study also had a few limitations. Firstly, the sample size of the data was limited. Although we had increased the sample size by including as many years as we can, the basic unit of observation was still province. Further studies might try to utilize data with smaller observation unit, such as data from the 336 surveillance spots (126 urban areas and 210 rural areas) in the National Maternal Mortality Surveillance System (NMMSS) of China. Moreover, this study only focused on whether the pregnant women received the required maternal health services or not, the service quality were not considered. With more comprehensive data on maternal health program management and assessment, the quality and utilization of the services may be considered separately. Finally, the maternal health services could be only one of the factors affecting maternal health conditions and maternal mortality, other crucial factors relating to maternal mortality such as the overall health service quality [36, 37] and emergency obstetric care [38] could not be measured and controlled in this study due to the lack of available data.

## Conclusion

This study found that the utilization of maternal health services from antenatal to postpartum period could be associated with the decrease in the MMR. The design and implementation of the maternal health service program of the BPHS project in China may offer an example for other developing countries that wish to improve maternal health and maternal health services. Further studies could take counties or individuals as sampling unit to enlarge the sample size. The different causes of maternal death should also be explored so that the association and even causal relationship between maternal health services and maternal death could be further analyzed.

## Data Availability

The datasets generated and analyzed during the current study are available in the official website of National Health Commission of the People’s Republic of China (http://www.nhc.gov.cn).

## Abbreviations

MMR: Maternal mortality ratio
MDG: Millennium Development Goals
SDG: Sustainable Development Goals
BPHS: Basic Public Health Service
NMMSS: National Maternal Mortality Surveillance System
WHO: World Health Organization
NCMS: New Cooperative Medical Scheme
